# Cellulitis of the Neck

**Published:** 1884-12

**Authors:** 


### Selections.

---

### CELLULITIS OF THE NECK.

The following is from a paper read before the Bengal branch of the British Medical Association, by G. Harrison Young, L. K. Q. C. P. I., etc.: Strange to say cellulitis of the neck, although a most important and dangerous affection, is not even mentioned in the ordinary text-books on surgery. Yet a glance at the anatomy of the neck is sufficient to show how important is this disease. When inflammation of the cellular tissue of the neck takes place, serum and pus are effused under the cervical fascia, giving rise to severe and dangerous pressure, not only on the larynx and trachea, pharynx and œsophagus, but also on the large blood-vessels passing to and from the brain, and on the sympathetic and pneumogastric nerves. As Mr. H. G. Croly, of Dub'in, says, when writing of this affection: "Diffuse inflammation of the areolar tissue of the neck is one of the most serious and fatal forms of disease which the erysipelatous type assumes."

Diseased states of the blood, especially those occurring in Bright's disease and in the gouty diathesis, predispose to erysipelas, and therefore to this disease. In many cases, however, the disease comes on suddenly in strong, healthy persons, without the previous existence of any of these conditions. The exciting cause is always exposure to wet and cold. The disease, therefore, almost always occurs in winter, and is most prevalent in low, damp situations. Cellulitis of the neck, unlike other erysipelatous diseases, is rarely, if ever, traumatic, and is, probably, never due to infection.

There is never any difficulty in diagnosing this affection. The brawny feel of the tissues, and the subsequent pitting on pressure, distinguish it from every other disease found in this region. In this disease, although there is abundant effusion of serum and pus, fluctuation is never obtained. This is due, I believe, to the fact that the effusions are always situated under the deep cervical fascia, which binds them down so firmly that we cannot detect fluctuation.

PATHOLOGY.

This disease consists essentially of an erysipelatous inflammation of the areolar tissue, which fills up the spaces between, and connects together the deep cervical muscles. This tissue rapidly breaks down, giving rise to sloughs, while at the same time serum and pus are effused into the intermuscular spaces. As nearly all this mischief takes place beneath the deep cervical fascia, which is dense and unyielding, we can at once see what an amount of pressure is exercised on the important anatomical structures of the neck, and how necessary it is to give relief to this pressure by free incisions.

TREATMENT.

Of all the diseases to which man is subject, none requires more prompt treatment than cellulitis of the neck; for if relief be not afforded by surgical means, the disease is almost necessarily fatal, or if recovery do chance to take place without operation, it will only be after sloughing has set in, and extensive disorganization of the muscles of the neck has taken place, which will give rise to stiffness and great deformity. From the first, our treatment should be such as to support the strength of the patient, and all remedies which tend to lower the powers of the system should be carefully avoided. It is to be remembered that the patient is suffering from a disease of a highly asthenic type, and will also have to pass through a period of prolonged suppuration before recovery takes place. Lowering treatment also tends to increase the chances of pyæmia occurring. The patient should have a good purgative at the commencement of the disease, as the bowels are always constipated. He should then be placed on a mixture containing chlorate of potash combined with tincture of the perchloride of iron, or nitric acid with decoction of cinchona. It seems to me that a combination of chlorate of potash with tincture of iron has a peculiar and most beneficial effect in all erysipelatous diseases, and I now invariably prescribe it. At the same time plenty of milk, beef-tea, chicken-broth and raw eggs and brandy should be given. Overstimlation, however, is to be carefully avoided, and the quantity of brandy given should be regulated by the amount of asthenia present. If the patient cannot swallow these articles they should be given in the form of enemata. If given thus, the benefit to the patient will be greatly increased by the addition to each enema of a little pepsine and hydrochloric acid about half an hour before it is administered. Local applications, as large linseed-poultices, hot fomentations, and poppy-stupes, should be applied, as they relieve pain and tension. However, all these measures are of small importance in comparison to the surgical treatment, as they have little or no curative power over the disease, and if they alone be used the patient will probably die. As Mr. H. G. Croly says: "The symptoms are urgent and alarming, and if relief be not afforded by prompt and bold surgical aid, the patient may be suffocated."

As soon as the brawny feeling of the tissues, with pitting on pressure, sets in, free and deep incisions should be made into the cervical fascia. When these incisions have been made, the relief which they give is immediate, even though no serum or pus escape; as the cervical fascia, being divided, stretches and tears, and the tension is thus greatly lessened. I have seen patients who had almost complete aphonia and dysphagia able to speak and swallow in five minutes after incisions had been made, and this even though no fluid escaped.

In making the incisions it is only necessary to remember the anatomy of the neck to do so with perfect safety.

Before concluding I would refer to a variety of this disease, first described by the late Dr. Osbrey, of Dublin, and which is nearly always fatal—namely, that occurring in children recovering from malignant scarlatina. In these cases the disease sets in very insidiously, and is invariably followed by sloughing. Dr. Osbrey believed it was not good practice to make incisions into the cervical fascia in these cases, as the patients are not able to bear them, and they, in his opinion, only hasten a fatal termination. These cases are to be treated with chlorate of potash and tincture of iron, together with all the nourishment the patient can be got to take.—*Brit. Med. Jour, Analectic.*

